# Innovative Horizons in Fall Risk Self-Management for Older Adults: An Integrative Review and Model

**DOI:** 10.1093/geroni/igaf122.2635

**Published:** 2025-12-31

**Authors:** Angela Shanahan, Patricia Groves, Harleah Buck

**Affiliations:** University of Iowa, Iowa City, Iowa, United States; University of Iowa College of Nursing, Iowa City, Iowa, United States; University of Iowa, Iowa City, Iowa, United States

## Abstract

Falls affect 1 in 4 community-dwelling older adults. Despite focusing on fall prevention factors such as knowledge about falls, mobility, and fear of falling, researchers and clinicians continue to report high fall rates and low adherence to prevention programs. The Health Belief Model (HBM) suggests unexplored factors (i.e. perceptions and beliefs), which may influence older adults’ engagement in fall risk self-management. The purpose of this review was to conduct a comprehensive review of the literature examining the relationships among HBM factors: perceptions/beliefs, cues-to-action, self-efficacy, and older adults’ fall risk self-management and create a conceptual model. Pubmed, PsychINFO and CINAHL were searched using the terms: perception, belief, motivation, fall risk, and community-dwelling. Inclusion criteria: English language and included perceptions or beliefs related to fall risk. Thirty studies comprised of quantitative, qualitative, and systematic reviews met the criteria. The majority of the articles (n = 23, 60.52%) were published within the last 10 years and conducted within the United States (n = 18, 60%). The average age of participants was 75.4 years. Few studies (n = 3) examine individual perceptions/beliefs directly as they relate to self-management. Concepts were abstracted to inform the conceptual model demonstrating how the HBM concepts can innovatively inform future studies. Understanding how an individual’s beliefs: a) perception of fall risk, b) perception of injury, c) belief in ability to self-manage risk, and d) perception of benefit vs. barriers influence d) cues-to-action, and e) self-efficacy is crucial for advancing the science and developing effective strategies to support older adults in engaging in fall risk self-management.

